# Efficient Treatment of Oily Sludge via Fast Microwave-Assisted Pyrolysis, Followed by Thermal Plasma Vitrification

**DOI:** 10.3390/molecules28104036

**Published:** 2023-05-11

**Authors:** Qinglong Xie, Zhen Chen, Yuqiang Zhou, Tongbo Pan, Ying Duan, Shangzhi Yu, Xiaojiang Liang, Zhenyu Wu, Weirong Ji, Yong Nie

**Affiliations:** Biodiesel Laboratory of China Petroleum and Chemical Industry Federation, Zhejiang Provincial Key Laboratory of Biofuel, College of Chemical Engineering, Zhejiang University of Technology, Hangzhou 310014, China

**Keywords:** oily sludge, microwave-assisted pyrolysis (MAP), pyrolysis kinetics, thermal plasma vitrification, heavy metals leaching

## Abstract

Oily sludge, as a critical hazardous waste, requires appropriate treatment for resource recovery and harmfulness reduction. Here, fast microwave-assisted pyrolysis (MAP) of oily sludge was conducted for oil removal and fuel production. The results indicated the priority of the fast MAP compared with the MAP under premixing mode, with the oil content in solid residues after pyrolysis reaching below 0.2%. The effects of pyrolysis temperature and time on product distribution and compositions were examined. In addition, pyrolysis kinetics can be well described using the Kissinger-Akahira-Sunose (KAS) and the Flynn-Wall-Ozawa (FWO) methods, with the activation energy being 169.7–319.1 kJ/mol in the feedstock conversional fraction range of 0.2–0.7. Subsequently, the pyrolysis residues were further treated by thermal plasma vitrification to immobilize the existing heavy metals. The amorphous phase and the glassy matrix were formed in the molten slags, resulting in bonding and, hence, immobilization of heavy metals. Operating parameters, including working current and melting time, were optimized to reduce the leaching concentrations of heavy metals, as well as to decrease their volatilization during vitrification.

## 1. Introduction

In the petroleum industry, a large amount of oily sludge is generated during the oil exploitation, storage, transportation, and refinery processes. Oily sludge usually exists as a complex semi-solid waste that is composed of water–oil emulsions mixed with solid particles [1]. In addition to the inorganic composition, which is similar to soil, the organic composition of oily sludge is mostly constituted of hydrocarbons, which are an oil resource. On the other hand, due to the toxicity, potential carcinogenicity, and mutagenicity of the existing heavy metals, oily sludge exhibits persistent environmental risks and is recognized as a critical hazardous waste in many countries [2]. Therefore, appropriate treatment of oily sludge is needed for both resource recovery and harmfulness reduction [3].

Currently, the main methods for oily sludge treatment include landfilling, demulsification [4], solvent extraction [5], biodegradation [6], incineration [7], and pyrolysis [8]. Landfilling occupies much land and treats the oily sludge inefficiently, since it does not recover oil resources, and it easily causes secondary pollution. Demulsification removes water and recovers the oil from the oily sludge. Yet, it has the disadvantages of large demulsifier consumption for chemical demulsification and large energy consumption for freeze/thaw demulsification [9]. Solvent extraction is a less energy-intensive method for oil recovery. Whereas, large amounts of organic extractant are needed, and the efficiency is relatively low [10]. Biodegradation requires low energy consumption and investment cost (with large processing capacity), but it usually needs a long treatment cycle [11]. Incineration of oily sludge can achieve significant reduction in volume. Yet, high energy consumption and severe secondary pollution (NO_X_, SO_X_, and heavy metals) limit its application [12]. Moreover, resource recovery during the incineration process is difficult, since most oil is completely converted into CO_2_ and H_2_O. Pyrolysis cracks the oils into high value-added liquid and gaseous fuels in the absence of oxygen, with little NO_X_ or SO_X_ formed. Additionally, most heavy metals remain in the solid product [11]. Therefore, pyrolysis is an effective and promising method for oily sludge treatment, which can be used to recover oil resources and, additionally, to reduce secondary pollution.

Compared with conventional heating, microwave irradiation as an alternative heating method offers many advantages, including fast and bulk heating, ease of set-up and operation, and low cost [13]. The microwave-assisted pyrolysis (MAP) technique has been applied in the treatment of various wastes and biomass feedstocks [14]. Liu et al. [15] conducted a MAP of oily sludge and obtained the highest oil yield of 85.93 wt% at a pyrolysis time of 15 min and a temperature of 500 °C. However, most studies on the MAP of oily sludge achieve heating and pyrolysis by premixing the feedstock with certain microwave-absorbing materials, which, to a certain extent, limits the heating rate and, hence, the pyrolysis efficiency [16]. By comparison, fast microwave-assisted pyrolysis first heats the microwave absorbent bed to a set temperature, and, then, fast pyrolysis occurs once the feedstock is introduced and contacts with the high-temperature bed [17]. The fast MAP is reported to enhance the production of pyrolysis oil [18,19]. Thus, the application of fast MAP in oily sludge treatment is expected to improve the resource recovery and, additionally, reduce the oil content and, hence, the harmfulness of the pyrolysis residues.

Although the problems of oil pollution and resource waste for oily sludge can be effectively solved through the pyrolysis process, the environmental risks caused by heavy metals still remain an issue. However, few studies on oily sludge treatment focused on the disposal of heavy metals [20]. Employing high-energy plasma, the technique of thermal plasma vitrification can effectively reduce the volume of and immobilize the heavy metals in hazardous wastes at relatively short melting time [21]. Thermal plasma furnaces have been applied in the treatment of various hazardous materials, such as radioactive wastes, municipal solid waste (MSW), and MSW incineration residues [22]. Wang et al. [23] adopted thermal plasma technology to vitrify the fly ash from MSW incinerators, and they found an obvious reduction in the leaching of heavy metals after vitrification. Thus, thermal plasma vitrification is expected to immobilize the heavy metals and further reduce the harmfulness of the solid residues after the pyrolysis of oily sludge.

In this study, fast MAP of oily sludge was conducted for resource recovery. The effects of heating rate, pyrolysis temperature, and time on product distribution, oil, and gas compositions were examined. The kinetic parameters for the oily sludge pyrolysis described by different kinetic models were calculated and compared. In addition, the solid residues after pyrolysis were further vitrified on a thermal plasma furnace. The effects of working current and melting time on the leaching of heavy metals were investigated.

## 2. Results and Discussion

### 2.1. Fast MAP of Oily Sludge

#### 2.1.1. Comparison between Fast MAP and MAP While Using Premixing Mode

**Figure 1:**
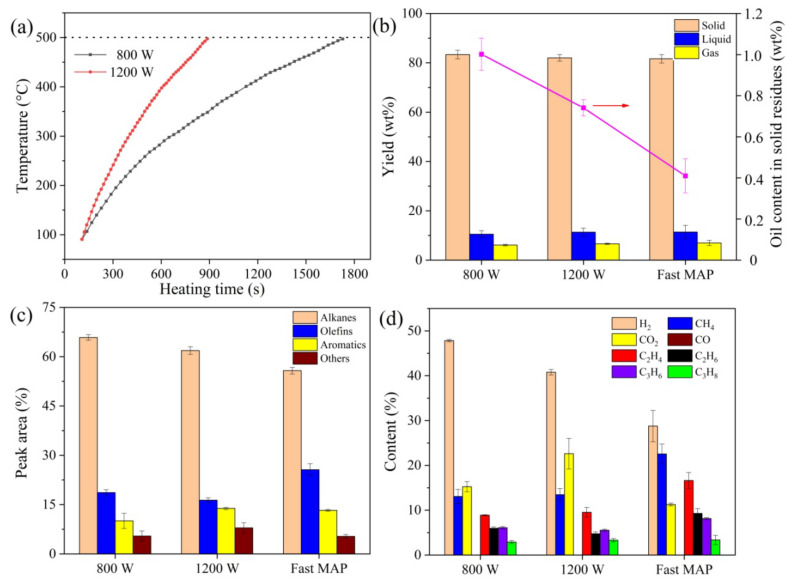
Comparison between fast microwave-assisted pyrolysis (MAP) and MAP while using the premixing mode for oily sludge pyrolysis. (**a**) Temperature profiles for MAP under premixing mode. (**b**) Product distribution and oil content in solid residues. (**c**) Composition of liquid product. (**d**) Composition of gas product.

To prove the advantages of fast microwave-assisted pyrolysis, the fast MAP of oily sludge was conducted and compared with the MAP tests performed under the premixing mode. As shown in Figure 1a, although the microwave irradiation could provide fast heating, a period for temperature increase was still needed for pyrolysis while using the premixing mode. The average heating rates at a microwave power of 800 W and 1200 W were 17.4 and 33.8 °C/min, respectively. By comparison, the instantaneous heating and pyrolysis of oily sludge can be achieved during the fast MAP process through the direct contact of the feedstock with the high-temperature silicon carbide (SiC) bed. As displayed in Figure 1b, compared with the premixing mode, a slight decrease in solid yield and an increase in gas and liquid yields were observed for fast MAP at 500 °C. More importantly, the oil content in the solid residues was significantly reduced through fast pyrolysis. This mainly occurred because of the higher heating rate, which favored oil production during the pyrolysis process [24,25]. Thus, the fast MAP technique for oily sludge treatment could enhance oil recovery and reduce the harmfulness of the solid residues.

In addition, as presented in Figure 1c,d, not much difference in the compositions of gas and liquid products was found for the two different MAP modes. Higher olefin production and lower alkane yield were obtained in the fast MAP of oily sludge than the premixing process. This was probably because, in fast MAP, the instantaneous contact with high-temperature SiC bed provided the feedstock with more energy [26], which promoted the conversion of alkanes as the main constituents of oil to olefins. Overall, compared to the premixing mode, the fast MAP is a more promising method for oily sludge treatment from the perspective of resource recovery and environmental protection.

#### 2.1.2. Effect of Temperature

**Figure 2:**
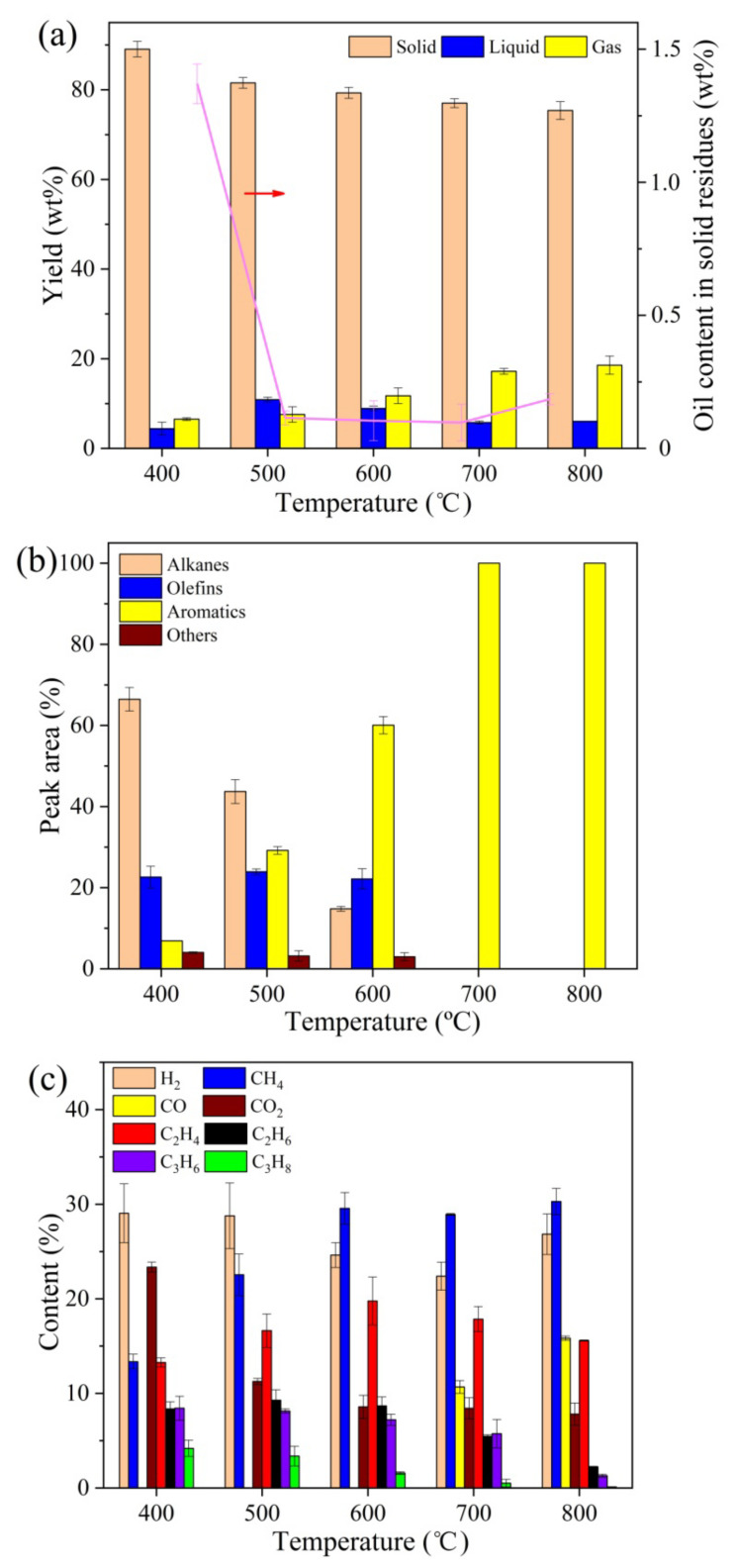
Effect of temperature on fast MAP of oily sludge at the pyrolysis time of 30 min. (**a**) Product distribution and oil content in solid residues. (**b**) Composition of liquid product. (**c**) Composition of gas product.

The effect of temperature on product distribution and compositions in the fast MAP of oily sludge at the pyrolysis time of 30 min is shown in Figure 2. A continuous decrease in solid yield and a continuous increase in gas production, with increasing temperature, can be clearly noticed in Figure 2a, with the liquid yield first increasing and then decreasing. Higher temperatures improved the devolatilization of oily sludge, which resulted in the decrease in solid yield and initial increase in liquid production with temperature. However, the thermal cracking of oil vapors into smaller molecules was obviously enhanced at a temperature higher than 500 °C because more energy was available to break the strong organic bonds. Consequently, the liquid yield decreased when more gases formed. In addition, the oil content in pyrolysis residues was reduced to below 0.2 wt% and changed slightly when the temperature was higher than 500 °C. From the perspective of oil removal, the temperature of 500 °C was adequate for the fast MAP of oily sludge. For the composition of liquid product, as displayed in Figure 2b, the bonds in alkanes and olefins were more easily broken at higher temperatures to form short-chain olefins and free radicals, which promoted the generation of aromatics [27,28]. Moreover, Figure 2c shows that higher temperatures favored the production of CH_4_ and CO at the expense of CO_2_ and other hydrocarbons, which was achieved probably through the hydrocarbons’ cracking and methane reforming reactions [29]. The results are in accordance with those in previous reports on various pyrolysis processes [30,31].

As presented in Figure 2b, aromatic hydrocarbons existed as the main components in the liquid product obtained at higher temperatures, which are utilized as important industrial chemicals and transportation fuel additives to increase octane number [32]. It is suggested that the solid part of oily sludge may serve as a catalyst for the conversion of alkanes and olefins into aromatics. The pathway for aromatic formation from olefins basically includes activation, cleavage, oligomerization, dehydrogenation, cyclization, and aromatization [33]. The catalytic performance towards aromatic production is greatly influenced by the surface acidity of a catalyst, especially the density of Brønsted acid sites (BAS) and Lewis acid sites (LAS) [34]. The results of Fourier-transform infrared spectroscopy of pyridine (pyridine-FTIR) analysis show that the amounts of weak, moderate, and strong BAS on the volatile extracted oily sludge were 29.6, 22.7, and 17.2 μmol/g, respectively, while those for LAS were 118.5, 77.5, and 50.5 μmol/g, respectively. Compared to BAS, more LAS, especially more strong LAS, could enhance the dehydrogenation reaction and inhibit hydrogen transfer, which would promote the aromatics’ formation [35,36]. Thus, the relatively high density of LAS and strong LAS on the oily sludge led to a high yield of aromatic hydrocarbons in the fast MAP process.

#### 2.1.3. Effect of Pyrolysis Time

**Figure 3:**
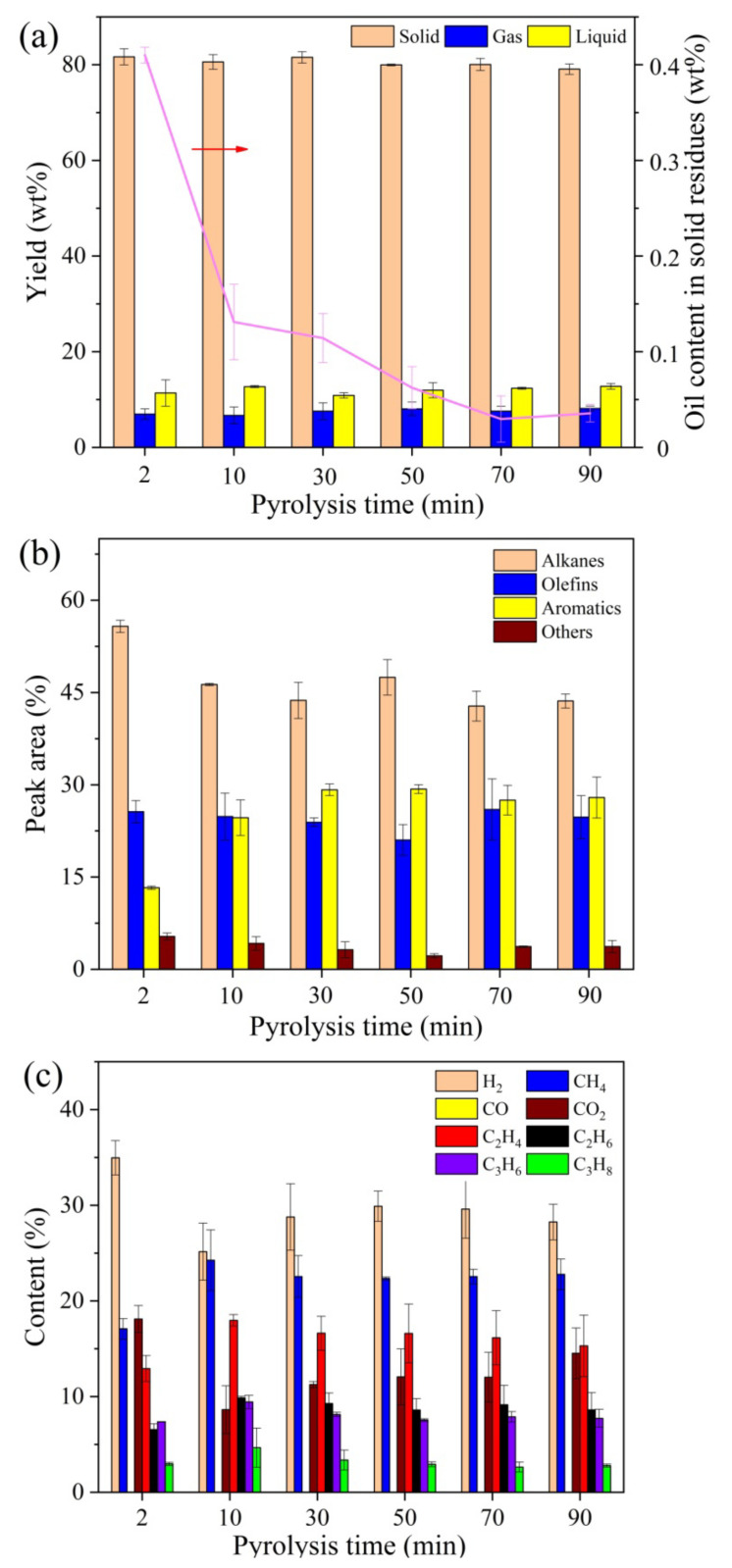
Effect of pyrolysis time on fast MAP of oily sludge at the pyrolysis temperature of 500 °C. (**a**) Product distribution and oil content in solid residues. (**b**) Composition of liquid product. (**c**) Composition of gas product.

The effect of pyrolysis time on product distribution and compositions in the fast MAP of oily sludge at the pyrolysis temperature of 500 °C is displayed in Figure 3. Overall, no significant differences in product distribution, or with regard to liquid or gas compositions with various pyrolysis time, were observed, especially when the pyrolysis time was longer than 10 min. This indicated that the pyrolysis process was basically completed within 10 min. The instantaneous heating and pyrolysis of feedstock contributes to the short reaction time in the fast MAP process. Compared with the MAP process under the premixing mode that requires much longer time consumption, the fast MAP with higher efficiency is more suitable for industrial applications.

### 2.2. Pyrolysis Kinetics

**Figure 4:**
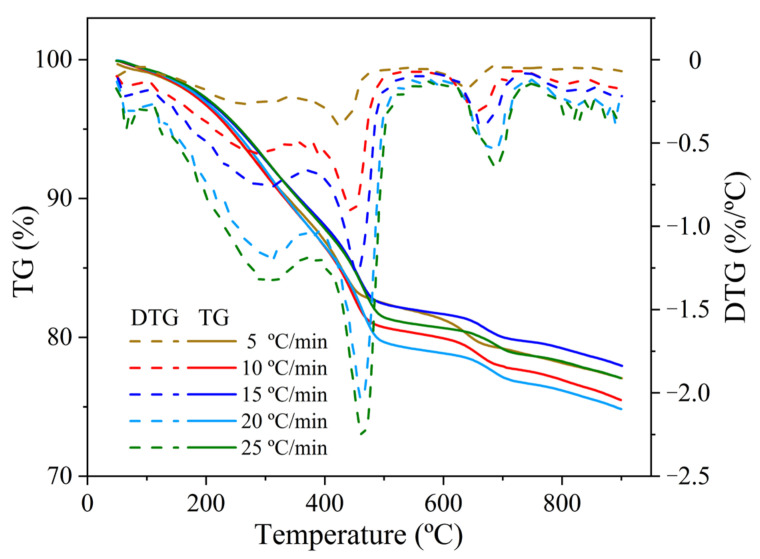
Thermogravimetric (TG) and derivative thermogravimetric (DTG) curves of the oily sludge feedstock.

**Figure 5:**
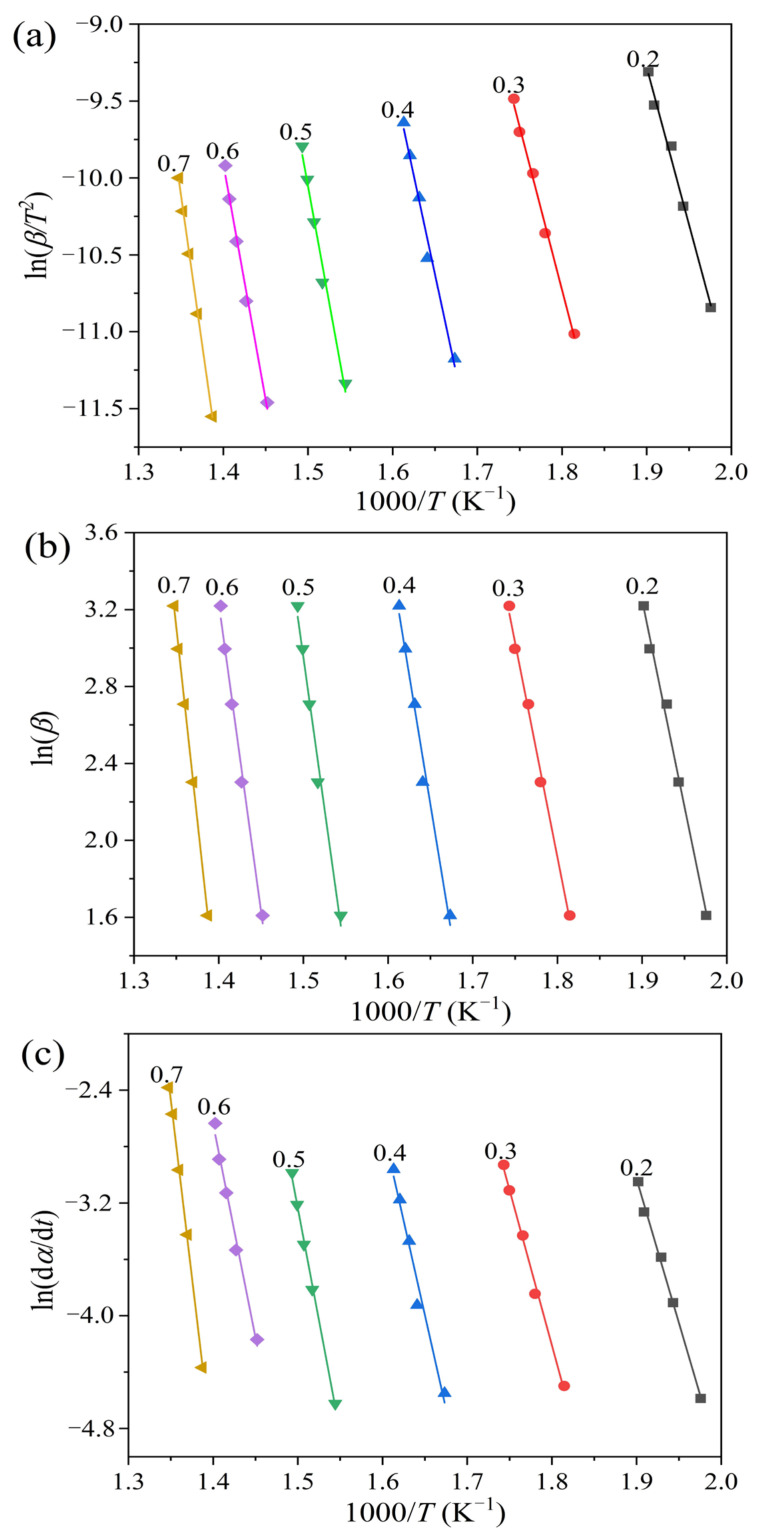
Activation energy plots for the (**a**) Kissinger-Akahira-Sunose (KAS), (**b**) Flynn-Wall-Ozawa (FWO), and (**c**) Friedman methods.

In this work, three model-free methods, i.e., the Kissinger-Akahira-Sunose (KAS), Flynn-Wall-Ozawa (FWO), and Friedman methods, were adopted to determine the kinetic parameters for the pyrolysis process of oily sludge. The thermogravimetric analysis (TGA) data at five different heating rates, as displayed in Figure 4, were used to fit the corresponding kinetic models described by Equations (5)–(7). Figure 5 shows plots of ln (*β*_i_/*T*_αi_^2^) versus 1/*T*_αi_, ln *β*_i_ versus 1/*T*_αi_, and ln (*dα*/*dt*) versus 1/*T*_αi_ for the KAS, FWO, and Friedman methods, respectively. The iso-conversional kinetics were evaluated in the feedstock conversional fraction range of 0.2–0.7. However, the conversional range of 0.3–0.7 was previously suggested in a kinetic study to avoid inaccuracy due to the derivative thermogravimetric (DTG) peak tail [37]. In fact, the error for kinetic evaluation at the conversional range of lower than 0.3 was mainly attributed to moisture evaporation [38]. Since the oil sludge feedstock used in this work has rather low moisture content, the pyrolysis kinetics were studied at the conversional range of 0.2, at which point a high correlation coefficient was still obtained.

Table 1 lists the apparent activation energies for oily sludge pyrolysis, which was calculated based on the fitting plots for the KAS, FWO, and Friedman methods. High correlation coefficients for all the activation energies were obtained, suggesting the accuracy of the calculated values. For all the three model-free methods, the activation energy of oily sludge pyrolysis continuously increases with an increasing feedstock conversional fraction. This indicates that the pyrolysis process is composed of complex reactions that involve multi-step mechanisms. The reactions occurring at higher temperatures usually require larger activation energies. From this perspective, these three model-free methods that analyze the kinetic parameters at different conversional fractions are more reliable than other methods, such as the Coats-Redfern and Kissinger methods, which consider the kinetics from the entire reaction [39]. The activation energy of oily sludge pyrolysis, calculated using the KAS and FWO methods, shows a similar range of between 170.0–318.5 kJ/mol and 169.7–319.1 kJ/mol, respectively, with larger values obtained using the Friedman method in the range of 169.8–419.0 kJ/mol. The difference in activation energy, determined by the Friedman method against the KAS and FWO methods, essentially occurs due to their respective mathematical models. The KAS and FWO methods belong to an integral approach based on heating rate, whereas the Friedman method adopts a differential method for analysis, which is based on conversion rate. Compared to the differential methods, the integral methods are more accurate because their results are less likely to be influenced by experimental errors [40]. Similar results can be found in a previous report on the kinetics of horse manure pyrolysis [41].

### 2.3. Thermal Plasma Vitrification of Pyrolysis Residues

#### 2.3.1. Effect of Working Current

**Figure 6:**
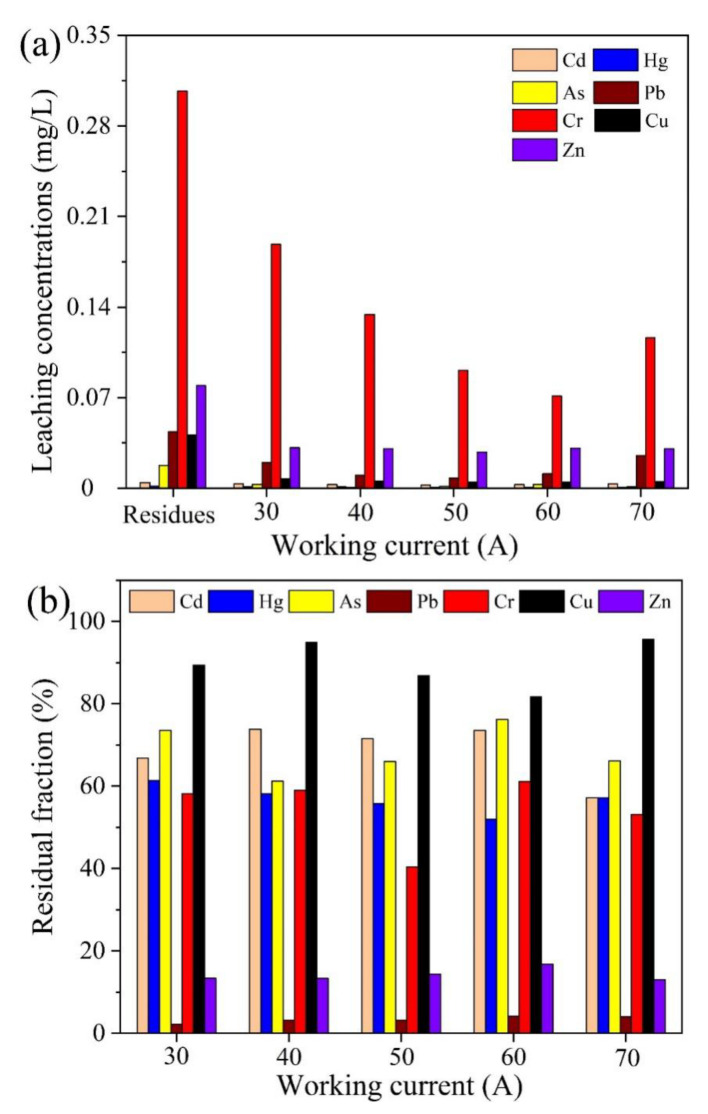
Effect of working current on thermal plasma vitrification of pyrolysis residues at a melting time of 20 min. (**a**) Leaching concentrations of heavy metals. (**b**) Residual fractions of heavy metals in molten slags.

Since the solid residues after the pyrolysis of oily sludge still contained various heavy metals that had potential environmental risks, the thermal plasma vitrification technique was adopted to immobilize the heavy metals. Figure 6 shows the effect of working current on the thermal plasma vitrification of pyrolysis residues at a melting time of 20 min. As displayed in Figure 6a, for all the working currents, the leaching concentrations of all the heavy metals in pyrolysis residues were significantly reduced after vitrification, and they were below the regulatory standard established by the US EPA. The results prove that thermal plasma vitrification is effective for immobilization of heavy metals. It was primarily attributed to the amorphous and glassy matrix formed within the slags that prevented the heavy metals from leaching [42].

**Figure 7:**
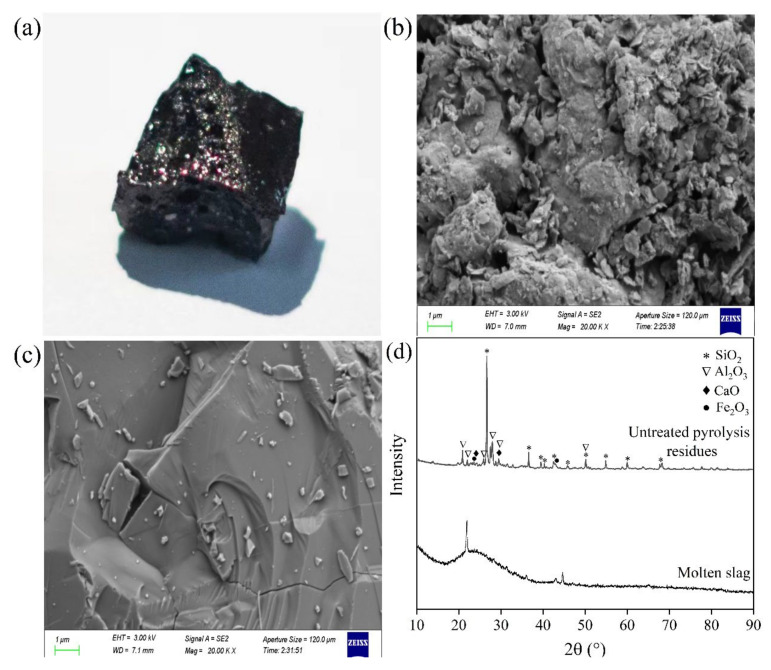
(**a**) Photograph of molten slag, scanning electron microscopy (SEM) micrographs of (**b**) untreated pyrolysis residues and (**c**) molten slag, and (**d**) powder X-ray diffraction (XRD) patterns of untreated pyrolysis residues and molten slag.

As presented in Figure 7a, the molten slag was vitreous and glazed in nature, with opaque and dark color appearance. Scanning electron microscopy (SEM) images (Figure 7b,c) show that the microstructure of pyrolysis residues was loose, with uneven particle size distribution, which was changed to be homogeneous and compact after thermal plasma treatment. More importantly, powder X-ray diffraction (XRD) patterns, as displayed in Figure 7d, demonstrated the generation of amorphous and glassy slags after vitrification, since almost all the crystalline peaks disappeared. The glass phase content in the slag, determined according to the GB/T 18046-2017 standard, reached up to 93.4%. Ions, such as Si^2+^, Al^3+^, and Ca^2+^ in the predominant phases of slags, could be substituted by heavy metal ions [43]. Thus, the heavy metals were strongly bonded in the glassy matrix, leading to a reduction in their leachability [44]. In addition, as shown in Figure 6a, the leaching concentrations of heavy metals decreased with increasing working current. Larger working current meant larger power and higher melting temperature, which favored the formation of an amorphous phase and glassy matrix [45]. Hence, this improved the immobilization of heavy metals.

Figure 6b presents the residual fractions of heavy metals in molten slags obtained at different working currents. All the heavy metals exhibited losses to certain extents, especially Pb and Zn, which were mainly due to their volatilization during vitrification [46]. Pb and Zn are generally considered to be easy volatile metals, Cu and Cd are considered to be medium volatile metals, and Cr is considered to be a low volatile metal [23]. Al_2_O_3_ existing in the pyrolysis residues could reduce the activity of CuO and, hence, greatly improve the stability of Cu in the thermal plasma treatment [47]. In fact, the volatilization of heavy metals was one important reason for the reduction in the leaching toxicity of molten slags. However, the evaporation of heavy metals into the air can cause secondary pollution that requires subsequent pollutant capture and treatment.

#### 2.3.2. Effect of Melting Time

**Figure 8:**
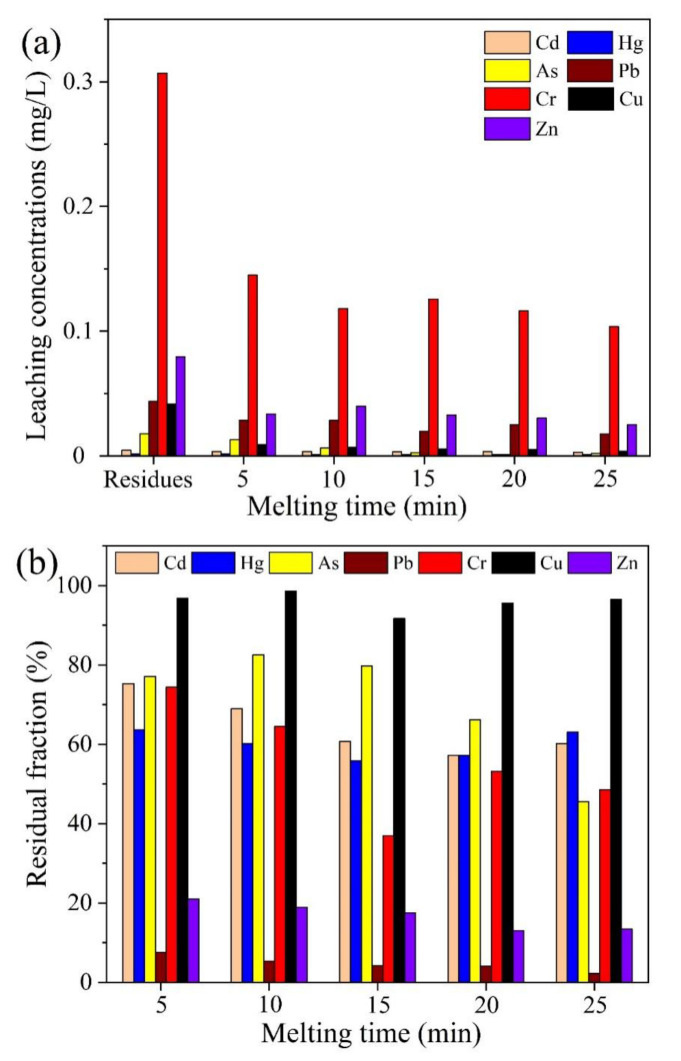
Effect of melting time on thermal plasma vitrification of pyrolysis residues at a working current of 70 A. (**a**) Leaching concentrations of heavy metals. (**b**) Residual fractions of heavy metals in molten slags.

The effect of melting time on the thermal plasma vitrification of pyrolysis residues at a working current of 70 A is shown in Figure 8. The leaching concentrations of all the heavy metals continuously decreased with the increase in melting time, which was mainly because the molten slags could become more amorphous and glassier with longer treatment time. However, as displayed in Figure 8b, more heavy metals would evaporate with increasing melting time, resulting in more serious air pollution. Considering both vitrification efficiency and heavy metal volatilization, the optimal melting time was around 10 min at a working current of 70 A. The molten slags that are stable and safe in the surroundings can be utilized as construction materials, such as road fill, concrete aggregate, and glass ceramics. Therefore, the technique of fast MAP, coupled with thermal plasma vitrification, is effective for oily sludge treatment, having achieved the objectives of resource recovery and harmfulness reduction.

## 3. Materials and Methods

### 3.1. Materials Preparation

The oily sludge samples were collected during the crude oil extraction on the Daqing Oilfield production platform, China. The results of proximate analysis obtained according to the ASTM D7582-2015 standard showed that moisture, as well as volatile and ash contents in the oily sludge, were 1.7 wt%, 21.8 wt%, and 74.4 wt%, respectively. The ultimate analysis was performed on a PerkinElmer 2400 Series II CHNS/O elemental analyzer, with the C, H, and N contents being 15.8 wt%, 2.9 wt%, and 0.11 wt%, respectively. Overall, the oily sludge samples used in this work have relatively low oil content.

### 3.2. Experimental Apparatus

#### 3.2.1. Fast MAP of Oily Sludge

**Figure 9:**
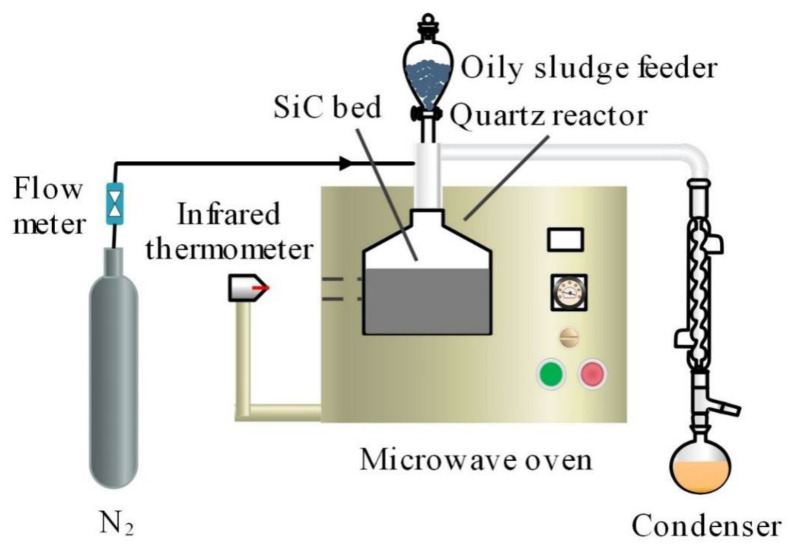
Experimental setup for fast microwave-assisted pyrolysis of oily sludge.

The fast MAP tests were performed in a self-designed microwave oven with a maximum power of 1200 W at a microwave frequency of 2450 MHz. The schematic diagram of the experimental apparatus is shown in Figure 9. For safety purpose, a microwave detector was used to monitor microwave leakage.

SiC particles, with large microwave absorbing capacity, were selected as the microwave absorbent beds. For each test, 750 g of SiC particles were added in a quartz reactor, which was then placed in the cavity of the microwave oven. In order to maintain an inert atmosphere for the pyrolysis reaction, N_2_ at a flow rate of 100 mL/min was introduced into the system during the entire heating and pyrolysis process. The temperature of the SiC bed quickly reached the set value after the microwave oven was started for heating. Subsequently, an oily sludge sample, in the amount of around 20 g, was dropped into the quartz reactor, and the pyrolysis reaction occurred once the sample contacted the hot bed. Meanwhile, the microwave oven was controlled to be on or off to maintain a stable temperature of the SiC bed, with the deviation of temperature being monitored by an infrared radiation thermometer within ±1 °C. The condensable components in the product vapor were condensed by the condensers and collected as the liquid product, with the non-condensable components collected as the gas product for subsequent analysis. The solid residues in the reactor after pyrolysis were separated from SiC particles and then collected as feedstock for the subsequent thermal plasma vitrification. The yields of liquid product and solid residues were determined on the basis of their actual weight, while the gas yield was calculated by differences based on the mass balance.

For comparison purposes, the MAP of oily sludge was also conducted while using the premixing mode. The experimental apparatus and process were basically the same, except that the oily sludge sample was fully mixed with the SiC particles before the start-up of the microwave oven. In addition, to remove the air within the reactor, the 100 mL/min N_2_ flow was introduced into the system prior to the commencement of microwave heating for 30 min.

#### 3.2.2. Thermal Plasma Vitrification of Pyrolysis Residues

**Figure 10:**
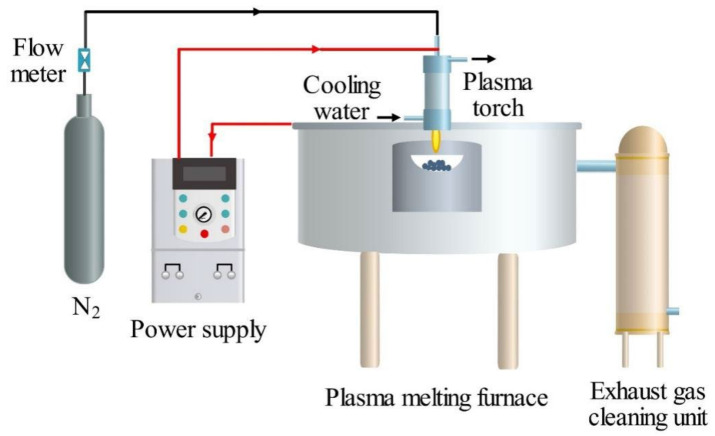
Experimental setup for thermal plasma vitrification of pyrolysis residues.

The vitrification of the solid residues after pyrolysis was carried out in a lab-scale 30 kW DC arc plasma furnace system. As shown in Figure 10, the system consisted of six parts, including a plasma melting furnace, a plasma torch, a power supply system (LGK-80 C, input voltage 380 V, output current 20–80 A), a working gas unit, a cooling water unit, and an exhaust gas cleaning unit. A solid residues sample, in the amount of around 20 g in a silica crucible, was placed inside the melting furnace, which was located at the same vertical position with the plasma torch that was installed at the top of the furnace. Nitrogen was adopted as the working gas at a flow rate of 1.2 m^3^/h, and running water at room temperature was used as the cooling water. The melting temperature was controlled by adjusting the working current of the DC arc plasma power supply. After a certain melting time, the molten slags were cooled and collected for subsequent analysis.

### 3.3. Product Analysis

The oil contents in the oily sludge feedstock and the solid residues after pyrolysis were determined according to HJ/T 970-2018 on a Lambda 35 UV/VIS spectrometer (PerkinElmer, Waltham, MA, USA). Petroleum ether was used as the solvent for oil extraction, with linear correlation between oil content and absorbance established for calculation. The acid properties of oily sludge with the oil extracted were assessed by the pyridine-FTIR technique, with the spectra recorded in a Nicolet 380 FTIR apparatus. The details can be found in our previous work [48].

The compositions of the extracted oil from the oily sludge and the liquid product from the pyrolysis process were analyzed using a 7890A–5975C gas chromatography/mass spectrometer (GC/MS) (Agilent, Santa Clara, CA, USA), which was equipped with a DB-5MS capillary column (30 m × 0.25 mm × 0.25 µm). Helium was used as the carrier gas at a flow rate of 50 mL/min. The injection size was 1 µL, with a split ratio of 1:20. The oven temperature was 70 °C initially, it was increased to 280 °C at a rate of 10 °C/min, and then it was increased to 305 °C at a rate of 5 °C/min. The temperature of the injector was maintained at 280 °C. The compounds were identified by comparing their mass spectra with those from the National Institute of Standards and Technology (NIST) mass spectral data library. Calibration was not carried out due to the large number of compounds in the oil samples. A semi-quantitative method was used to determine the relative proportion of each compound in the oil by calculating the chromatographic area percentage. The composition of gas products from the pyrolysis process was determined using two gas chromatographs (GC), with the details referring to our previous report [49].

TGA performed in a TG 209 F3 Tarsus simultaneous thermal analyzer (Netzsch, Selb, Germany) was used to investigate the pyrolysis characteristics of oily sludge. Nitrogen provided by a pressured tank at a flow rate of 250 mL/min was used as the carrier gas. The baseline was subtracted from a blank run. An oily sludge sample of around 8 mg was loaded into a hermetically sealed alumina crucible with a pinhole lid. The measurements were carried out at room temperature to 900 °C at heating rates of 5, 10, 15, 20, and 25 °C/min, respectively. The profiles of weight loss during the heating period were automatically recorded and plotted as a function of temperature in TG and DTG formats.

The morphology of pyrolysis residues before and after thermal plasma vitrification was observed by SEM technique. The SEM images were acquired on ZEISS Sigma 300 equipment, with the accelerating voltage set to 3 kV. The XRD technique was used to identify the major crystalline phases and the crystallinity present in the pyrolysis residues and molten slags. The XRD patterns were obtained on a Ultima IV X-ray diffractometer instrument (Rigaku, Tokyo, Japan), having Cu–Kα radiation at 40 kV and 40 mA. Data collected from the instrument were analyzed using the software MDI Jade 6.5.

To determine the concentrations of major heavy metals (Cd, Hg, As, Pb, Cr, Cu, and Zn) in the pyrolysis residues and molten slags, the samples were first digested according to the ASTM D5513 standard. The heavy metal concentrations were then measured on a PinAAcle 900F atomic absorption spectrometer (AAS) (PerkinElmer, Waltham, MA, USA). The leaching behavior of these heavy metals was investigated according to the EPA TCLP 1311 method. The leaching concentrations were calculated on the basis of the analysis results obtained on the AAS.

### 3.4. Kinetic Study of Oily Sludge Pyrolysis

The kinetics for the pyrolysis processes can be described by the following equation [50]:

(1) $$\frac{d\alpha }{dt}=k\left(T\right)\cdot f\left(\alpha \right)$$

where *α* is the immediate feedstock conversional fraction during pyrolysis, which is defined as follows:

(2) $$\alpha =\frac{m_{0}-m_{t}}{m_{0}-m_{f}}$$

where *m*_0_, *m*_t_, and *m*_f_ denote the initial mass of feedstock, feedstock mass at time t, and the final mass of solid residues after pyrolysis, respectively. Then, *f*(*α*) is a function that reflects the dependence of conversion on the apparent reaction progress.

The reaction rate constant in Equation (1), *k*(*T*), follows the Arrhenius equation:

(3) $$k(T)=A\exp\left(\frac{-E_{\alpha }}{RT}\right)$$

where *A* is the pre-exponential factor, *E*_α_ is the apparent activation energy, *R* is the universal gas constant, and *T* is the absolute temperature.

Integrating Equation (1), we obtain

(4) $$g\left(\alpha \right)=\int_{0}^{\alpha }\frac{d\left(\alpha \right)}{f\left(\alpha \right)}=\frac{A}{\beta }\int_{T_{0}}^{T}\exp(\frac{-E_{\alpha }}{RT})dT$$

where the function *g*(*α*) is the integral form of *f*(*α*), and *β* is the heating rate.

Equation (4) can be solved by a model-free method without considering the specific expression of the mechanism function *f*(*α*) [38]. This type of method belongs to an iso-conversional approach, in which the reaction rate at a specific conversional level for a certain heating rate is only a function of temperature, while *f*(*α*) remains unchanged. Typical iso-conversional model-free methods include the KAS, FWO, and Friedman methods [51,52].

#### 3.4.1. The Kissinger-Akahira-Sunose (KAS) Method

The KAS method transforms Equation (4) into the following equation without considering the mechanism function, *f*(*α*):

(5) $$\ln\left(\frac{\beta_{i}}{T_{\alpha i}^{2}}\right)=\ln\left(\frac{A_{\alpha }R}{E_{\alpha }g\left(\alpha \right)}\right)-\frac{E_{\alpha }}{RT_{\alpha i}}$$

From Equation (7), plotting ln (*β*_i_/*T*_αi_^2^) versus 1/*T*_αi_, a straight line with a slope of (–(*E*_α_*/R*)) can be obtained, and then the value of *E*_α_ can be calculated.

#### 3.4.2. The Flynn-Wall-Ozawa (FWO) Method

The FWO method integrates Equation (4) and implements Doyle’s approximation ($P_{D}\left(u\right)=0.0048e^{-1.0516u}$) to the temperature integral, and then it is rearranged to obtain:

(6) $$\ln\left(\beta_{i}\right)=\ln\left(\frac{A_{\alpha }R}{E_{\alpha }g\left(\alpha \right)}\right)-5.331-1.052\frac{E_{\alpha }}{RT_{\alpha i}}$$

The activation energy (*E*_α_) can be then calculated from the slope of the straight line plotted using the pairs of ln *β*_i_ and 1/*T*_αi_ data points.

#### 3.4.3. The Friedman Method

The Friedman method is derived from Equation (1) by taking the natural logarithm on both sides, which is expressed as follows:

(7) $$\ln{\left(\frac{d\alpha }{\mathrm{dt}}\right)}_{\alpha i}=\ln\left[A_{\alpha }\times f\left(\alpha \right)\right]-\frac{E_{\alpha }}{{\mathrm{RT}}_{\alpha i}}$$

Considering that the mechanism function *f*(*α*) remains constant, the value of *E*_α_ can be determined by fitting ln (*dα*/*dt*)_αi_ versus 1/*T*_αi_ to a linear graph with a slope of (–(*E*_α_*/R*)).

## 4. Conclusions

In this study, the technique of fast MAP, coupled with thermal plasma vitrification, was developed for the efficient treatment of oily sludge. Compared with MAP under premixing mode, more effective oil removal and fuel recovery were achieved in the fast MAP process. More LAS than BAS existed in the oily sludge, which resulted in aromatics as the main components in the liquid product at higher pyrolysis temperatures. The oil content in solid residues reached below 0.2 wt% at a temperature higher than 500 °C and a pyrolysis time longer than 10 min. Kinetic models established based on the model-free methods can be used to well describe the kinetics of oily sludge pyrolysis. The activation energy of the pyrolysis process was in the range of 170.0–318.5 kJ/mol and 169.7–319.1 kJ/mol, which was calculated using the KAS and FWO methods, respectively. The pyrolysis residues were then vitrified on a thermal plasma furnace. The leaching concentrations of all the heavy metals were significantly reduced after vitrification. The formation of amorphous and glassy molten slags favored the immobilization of heavy metals. From the perspective of vitrification efficiency and heavy metal volatilization, the optimal melting time was around 10 min at a working current of 70 A. Overall, the proposed technique is effective and efficient for oily sludge treatment to achieve the objectives of resource recovery and harmfulness reduction. Future research can focus on energy analysis and economic evaluation for the industrial applications of this technique.

## Figures and Tables

**Table 1:** Activation energy of oily sludge pyrolysis was obtained using the Kissinger-Akahira-Sunose (KAS), Flynn-Wall-Ozawa (FWO), and Friedman methods.

α	KAS Method		FWO Method		Friedman Method	
	E_α_ (kJ/mol)	R^2^	E_α_ (kJ/mol)	R^2^	E_α_ (kJ/mol)	R^2^
0.2	170.0	0.9919	169.7	0.9927	169.8	0.9966
0.3	176.2	0.9948	176.3	0.9953	183.3	0.9949
0.4	213.4	0.9857	212.4	0.9869	221.9	0.9762
0.5	251.8	0.9871	249.7	0.9881	265.0	0.9975
0.6	255.0	0.9924	253.5	0.9930	287.2	0.9890
0.7	318.5	0.9996	319.1	0.9996	419.0	0.9987

## Data Availability

Not applicable.
